# Supplementary material for: Systematic review of food insecurity and violence against women and girls: Mixed methods findings from low- and middle-income settings
Source: PLOS Glob Public Health. 2022 Sep 14;2(9):e0000479. doi: 10.1371/journal.pgph.0000479 (PMC10021293; doi:10.1371/journal.pgph.0000479)
Supplement: S1 Text — (DOCX) [file pgph.0000479.s001.docx]

Supplemental Appendix A

Search Strategy

PubMed

1. Food security

(“Diet, food, and nutrition”[mesh] OR “Food assistance”[mesh] OR “Food quality”[mesh]) OR (famine[Title/Abstract] OR “food availability”[Title/Abstract] OR “food poverty”[Title/Abstract OR “food insecurity”[Title/Abstract] OR “food insufficiency”[Title/Abstract] OR “food shortage”[Title/Abstract] OR “food sufficiency”[Title/Abstract] OR “food security”[Title/Abstract] OR hunger [Title/Abstract] OR hungry [Title/Abstract] OR starvation [Title/Abstract]) NOT (“eating disorders” OR bulimia OR anorexia OR dieting)

1. Violence against women and girls

"intimate partner violence"[mesh] OR domestic violence[mesh:noexp] OR "spouse abuse"[MeSH Terms] OR "battered women"[MeSH Terms] OR "intimate partner violence"[Title/Abstract] OR "gender based violence"[Title/Abstract] OR "partner violence"[Title/Abstract] OR "relationship violence"[Title/Abstract] OR "relationship aggression"[Title/Abstract] OR "couple violence"[Title/Abstract] OR "domestic violence"[Title/Abstract] OR "marital violence"[Title/Abstract] OR "spousal violence"[Title/Abstract] OR "partner abuse"[Title/Abstract] OR "relationship aggression"[Title/Abstract] OR "domestic abuse"[Title/Abstract] OR "marital abuse"[Title/Abstract] OR "spousal abuse"[Title/Abstract] OR "spouse abuse"[Title/Abstract] OR "wife beating"[Title/Abstract] OR "intimate terrorism"[Title/Abstract] OR "marital rape"[Title/Abstract] OR "battered women"[Title/Abstract] OR "abused women"[Title/Abstract] OR "rape"[Title/Abstract] OR "psychological abuse"[Title/Abstract] OR "reproductive coercion"[Title/Abstract] OR "violence"[Title/Abstract]

1. Not plants

(honey[Title/Abstract] OR seed*[Title/Abstract] OR plant*[Title/Abstract])

1. LMIC

Web of Science

1. Food security

TS=(famine OR food availability OR food poverty OR food insecurity OR food insufficiency OR food shortage OR food sufficiency OR food security OR hunger OR hungry OR starvation OR “Diet, food, and nutrition” OR “Food assistance” OR “Food quality”) NOT ALL=(eating disorders OR bulimia OR anorexia OR dieting)

1. Violence against women and girls

TS= (intimate partner violence OR partner abuse OR gender based violence OR relationship violence OR relationship aggression OR couple violence OR domestic violence OR marital violence OR spousal violence OR spousal abuse OR wife beating OR intimate terrorism OR marital rape OR battered women OR abused women OR reproductive coercion OR psychological abuse OR sexual abuse OR sex offense  OR "Partner Abuse" OR "Domestic Violence" OR "Battered Females" OR "Emotional Abuse" OR "Family Conflict" OR "Marital Conflict" OR "Partner Abuse" OR "Physical Abuse" OR "Sexual Abuse")

1. Not plants

TS=(honey OR seed* OR plant*)

1. LMIC

PsychInfo

1. Food security

TX (famine OR food availability OR food poverty OR food insecurity OR food insufficiency OR food shortage OR food sufficiency OR food security OR hunger OR hungry OR starvation) OR MA (“Diet, food, and nutrition” OR “Food assistance” OR “Food quality”) NOT TX (eating disorders OR bulimia OR anorexia OR dieting)

1. Violence against women and girls

AB (intimate partner violence OR partner abuse OR gender based violence OR relationship violence OR relationship aggression OR couple violence OR domestic violence OR marital violence OR spousal violence OR spousal abuse OR wife beating OR intimate terrorism OR marital rape OR battered women OR abused women OR reproductive coercion OR psychological abuse OR sexual abuse OR sex offenses)  OR DE ("Partner Abuse" OR "Domestic Violence" OR "Battered Females" OR "Emotional Abuse" OR "Family Conflict" OR "Marital Conflict" OR "Partner Abuse" OR "Physical Abuse" OR "Sexual Abuse")

1. Not plants

AB (honey OR seed* OR plant* OR crop)

1. LMIC
