# Supplementary material for: Systematic review of food insecurity and violence against women and girls: Mixed methods findings from low- and middle-income settings
Source: PLOS Glob Public Health. 2022 Sep 14;2(9):e0000479. doi: 10.1371/journal.pgph.0000479 (PMC10021293; doi:10.1371/journal.pgph.0000479)
Supplement: S1 Fig — (DOCX) [file pgph.0000479.s002.docx]

Supplemental Appendix B 1

Figure B.1. Meta-analysis of cross-sectional studies of relationship between food security and violence against women (by sex)

Appendix B.2

Figure B.2. Meta-analysis of cross-sectional studies of relationship between food security and violence against women (by VAWG type)

Appendix B.3

Figure B.3. Meta-analysis of cross-sectional studies of relationship between food security and violence against women (by region)

Appendix B.4

Figure B.4. Meta-analysis of cross-sectional studies of relationship between food security and violence against women (by timeframe)
