# Supplementary material for: Systematic review of food insecurity and violence against women and girls: Mixed methods findings from low- and middle-income settings
Source: PLOS Glob Public Health. 2022 Sep 14;2(9):e0000479. doi: 10.1371/journal.pgph.0000479 (PMC10021293; doi:10.1371/journal.pgph.0000479)
Supplement: S2 Fig — (DOCX) [file pgph.0000479.s003.docx]

Appendix C

Figure C. Funnel plot with 95% confidence intervals
