# Supplementary material for: Systematic review of food insecurity and violence against women and girls: Mixed methods findings from low- and middle-income settings
Source: PLOS Glob Public Health. 2022 Sep 14;2(9):e0000479. doi: 10.1371/journal.pgph.0000479 (PMC10021293; doi:10.1371/journal.pgph.0000479)
Supplement: Attachment — Submitted filename: Author response.docx [file pgph.0000479.s005.docx]

Dear PLoS Global Public Health,

We appreciate the input from editors and reviewers on our manuscript, “Systematic review of food insecurity and violence against women and girls: mixed methods findings from low- and middle-income settings.”

Please find, below, a point-by-point responses to the revision requests.

Very best,

Authors

Journal Requirements:

1. Please amend your online Financial Disclosure statement. If you did not receive any funding for this study, please simply state: “The authors received no specific funding for this work.”

*We revised the online statement.*

2. Please update your online Competing Interests statement. If you have no competing interests to declare, please state: “The authors have declared that no competing interests exist.”

*We revised the online statement.*

3. In the online submission form, you indicated that “Data can be accessed by contacting the authors.”. All PLOS journals now require all data underlying the findings described in their manuscript to be freely available to other researchers, either 1. In a public repository, 2. Within the manuscript itself, or 3. Uploaded as supplementary information.
This policy applies to all data except where public deposition would breach compliance with the protocol approved by your research ethics board. If your data cannot be made publicly available for ethical or legal reasons (e.g., public availability would compromise patient privacy), please explain your reasons by return email and your exemption request will be escalated to the editor for approval. Your exemption request will be handled independently and will not hold up the peer review process, but will need to be resolved should your manuscript be accepted for publication. One of the Editorial team will then be in touch if there are any issues.

*In alignment with this policy, all quantitative data are included within the manuscript in Table 1 and Figure 2. Qualitative data are found in the original manuscripts, which are all available online.*

4. Please provide separate figure files in .tif or .eps format and remove any figures embedded in your manuscript file. Please also ensure that all files are under our size limit of 10MB.

For more information about how to convert your figure files please see our guidelines: <https://journals.plos.org/globalpublichealth/s/figures>

*These have now been provided separately.*

5. We notice that your supplementary materials are included in the manuscript file. Please remove them and upload them with the file type ‘Supporting Information’. Please ensure that each Supporting Information file has a legend listed in the manuscript after the references list.

*We have added a legend in the manuscript and removed Supplemental information.*

6. Please review your reference list to ensure that it is complete and correct. If you have cited papers that have been retracted, please include the rationale for doing so in the manuscript text, or remove these references and replace them with relevant current references. Any changes to the reference list should be mentioned in the rebuttal letter that accompanies your revised manuscript. If you need to cite a retracted article, indicate the article’s retracted status in the References list and also include a citation and full reference for the retraction notice.

*The reference list is complete and correct.*

5. Review Comments to the Author

Please use the space provided to explain your answers to the questions above. You may also include additional comments for the author, including concerns about dual publication, research ethics, or publication ethics. (Please upload your review as an attachment if it exceeds 20,000 characters)

Reviewer #1: Paper is well written and follows the standard on systematic reviews writing. Authors have begun a discussion that could spur further discussion in the health arena especially within the feminist theorists.

*We are grateful to the reviewer for these comments.*

Reviewer #2: 1. The findings will show more relevant data if all the tools and resources are more clarified.

*We have included the tools used for measurement throughout the studies and would be happy to add further details if valuable? We did try to go beyond what is typically done in systematic reviews to offer precision around tools in the text:*

“Overall, measurement of food insecurity and VAWG in included studies was of high quality. Most studies used self-reported measures to assess food insecurity (with one study objectively assessing malnutrition by measuring body mass index [17]). All studies used self-reported measures of VAWG. While self-reports represent the state of art for food insecurity and VAWG research, there are clear limitations in reporting bias for this type of measure, such as underreporting and social desirability bias.

Multiple studies used comprehensive measures of food security. The Household Food Insecurity Access Scale (HFIAS) and the Brazilian Food Insecurity Scale (BFIS) measure constructs of quality and quantity of food supply as well as anxiety around food supply. Four studies used HFIAS [18-21] and two used BFIS [22, 23]. Another strong measure is the USDA Household Food Security Scale [24]. Three papers used 3-items of HFIAS related to household hunger (called the Household Hunger Scale [HHS]) [12, 25, 26]. Several studies used a single item to assess food insecurity [27-31], which may increase the risk of misclassification.

Measurement of VAWG varied but a majority used validated scales that assessed between 9 to 14 behaviourally-specific actions to classify whether violence occurred or not. Tools included the World Health Organization multi-country study instrument [12, 20, 24-26, 29, 30, 32-35], the Revised Conflict Tactics Scale [17, 22, 23, 27, 36, 37], the Sexual Experiences Survey [33], and the Demographic Health Survey Domestic Violence module [18]. Three studies used non-validated 1 to 3 single item questions asking participants whether they experienced any physical or sexual intimate partner? violence [21, 28, 31].”

2. In addition, can it enlist how the violence was minimized or counseled after the data collection in detail?

*While it is beyond the scope of this review to detail the ethical plans of each individual study, we agree that this would be important for the field and have added the topic to the limitations:*

“We did not abstract information from the studies around research ethics, but given the sensitive nature of the research topics this might be an important area for future reviews to examine more closely.”

3. Also could you add strategic plan in minimizing food insecurity and violence in recommendation?

*Good idea, we have added this to the Discussion:*

“Evidence for efficacious strategies that jointly target food insecurity and VAWG prevention is limited. This gap is crucial to fill, since these two social drivers of health are highly prevalent in many LMIC communities. Multiple programming approaches have the potential to be sustainable, scalable and cost-effective, but these have limited research demonstrating their efficacy. For example, Gender Action and Learning, a community-based methodology adopted by large-scale organizations such as Oxfam and IFAD, adds gender reflections and visioning exercises to agricultural and financial planning trainings. Gender Champions, a program implemented by Catholic Relief Services, conducts three home visits and implements ‘safe spaces’ where women and men can discuss common issues. Social Analysis and Action in Food and Nutrition Security, implemented by Care in more than 20 countries, asks community members to take practical steps to address gender, discrimination, or social norms through analysis-action-reflection. According to qualitative or anecdotal evidence, each of these programs seem to improve attitudes about VAWG and decision-making for women, lead to more equitable household division of labour, and offer better economic opportunities. However, none of these has been tested rigorously using experimental study designs, highlighting the importance of funding for research around food and safer relationships programming.

It terms of strategies going forward, programming should be framed as focused on family well-being to benefit households (rather than programming to transform gender). Women’s empowerment should be an implicit strategy of the intervention, even if this is not necessarily highlighted during community onboarding. Additionally, improving networks and social ties could be a promising strategy for promoting resilience for both food and violence prevention. Existing innovations in the field should be accompanied by rigorous evidence generation. This can be accomplished by adding VAWG and food security to ongoing trials, carefully evaluating scale-up activities in quasi-experimental design, and funder requirements for practice-based research that includes effectiveness findings.”

Reviewer #3: 1. I would disagree with using IPV instead of VAWG in the abstract because IPV is a type of the broad terminology of GBV (or VAWG as expressed by the author). I would recommend using VAWG instead of IPV.

*We agree and have edited the abstract to use the term VAWG throughout.*

- It would be more relevant to add a paragraph to the introduction about VAWG in LMIC as the study aims to estimate the effect of the association between food insecurity and VAWG in LMIC.

*This is a good point. We have added this paragraph:*

*“The World Health Organization estimates that globally, one in four women experience violence against women (VAWG) in their lifetime [1]. In sub-Saharan Africa, 44% of women experience VAWG from an intimate partner and one in six experience non-partner violence [2]. VAWG forms a persistent health crisis, leading to a high burden of injury, mental illness, physical decline, and mortality [3, 4]. VAWG is also a human rights travesty that circumvents governmental and bilateral commitments and undermines Sustainable Development Goals [5].”*

6. PLOS authors have the option to publish the peer review history of their article ([what does this mean?](https://journals.plos.org/globalpublichealth/s/editorial-and-peer-review-process#loc-peer-review-history)). If published, this will include your full peer review and any attached files.


**Do you want your identity to be public for this peer review?** If you choose “no”, your identity will remain anonymous but your review may still be made public.


For information about this choice, including consent withdrawal, please see our [Privacy Policy](https://www.plos.org/privacy-policy).

Reviewer #1: **Yes:** Ajike Saratu O

Reviewer #2: No

Reviewer #3: No
